# Laryngeal Edema and Dysphonia After the Use of Nasal Steroid Rinses

**DOI:** 10.7759/cureus.79954

**Published:** 2025-03-03

**Authors:** Jasmine Stewart, Austin Anderson, Amy Rutt

**Affiliations:** 1 Department of Otolaryngology, New York Institute of Technology College of Osteopathic Medicine at Arkansas State, Jonesboro, USA; 2 Department of Otolaryngology-Head and Neck Surgery, Mayo Clinic Florida, Jacksonville, USA

**Keywords:** allergic rhinitis, dysphonia, edema, laryngeal, sinusitis

## Abstract

Chronic sinusitis and allergic rhinitis are common conditions characterized by the inflammation of the nasal and sinus mucosa. While traditionally associated with symptoms such as nasal congestion and facial pain, recent research has shown that these disorders can also have a broader impact on vocal function. These symptoms are usually managed with intranasal corticosteroid (INCS) rinses or systemic corticosteroids. We present a case of a 60-year-old man with a history of chronic, recurrent sinusitis in which the traditional treatment with intranasal corticosteroid rinses caused dysphonia, dysphagia, and laryngeal edema. After multiple clinic visits and the cessation of the rinses, the patient's symptoms resolved entirely.

## Introduction

Chronic sinusitis, affecting over 28.9 million Americans, is characterized by the inflammation of the nasal and paranasal sinuses resulting in symptoms such as nasal discharge, nasal obstruction, facial pain/pressure, and/or the loss of taste/smell lasting 12 weeks or longer [1]. Standard care for recurring and allergic sinusitis includes saline irrigations and intranasal corticosteroids (INCS). In chronic rhinosinusitis (CRS), INCS reduce mucosal inflammation, inhibit cytokine release, and alleviate nasal congestion by directly targeting the nasal passages, but their limited distribution may reduce effectiveness in severe cases. Nasal steroid irrigation, by delivering steroids via a large-volume saline rinse, enhances drug penetration into the sinuses, improves mucociliary clearance, and is particularly beneficial for refractory CRS or postsurgical patients [2]. These treatments, when used consistently, have been shown to improve patients' quality of life (QoL) [2,3]. Bhattacharyya highlights the substantial impacts of CRS on QoL, including functional limitations, lost workdays, and negative effects on social behaviors [3].

Laryngeal edema can significantly increase morbidity and mortality if not accurately diagnosed and treated. Common etiologies include infections, trauma (such as intubation), medication adverse effects, allergic reactions, and C1 esterase inhibitor deficiency [4,5]. Patients may present with symptoms such as stridor, dysphonia, dysphagia, and dyspnea ranging from mild to severe, while others may remain asymptomatic depending on the underlying cause. The pharmacologic management of laryngeal edema aims to reduce inflammation and relieve associated symptoms, often by targeting the underlying sinonasal inflammation. Intranasal or nasal rinse corticosteroids, such as budesonide, fluticasone, and mometasone, help decrease mucosal swelling, improve drainage, and mitigate postnasal drainage that can contribute to persistent laryngeal irritation [1,2].

In this case report, we present a patient with dysphonia and a history of chronic, recurrent sinusitis. Despite improvement in nasal symptoms, the patient experienced worsening upper airway symptoms. Interestingly, the symptoms resolved after discontinuing steroid rinses, emphasizing the importance of closely monitoring treatment responses and adjusting therapeutic approaches when necessary. This case highlights the potential for nasal-steroid-rinse-induced laryngeal edema and underscores the need for further evaluation of optimal therapeutic strategies.

## Case presentation

**Figure 1:**
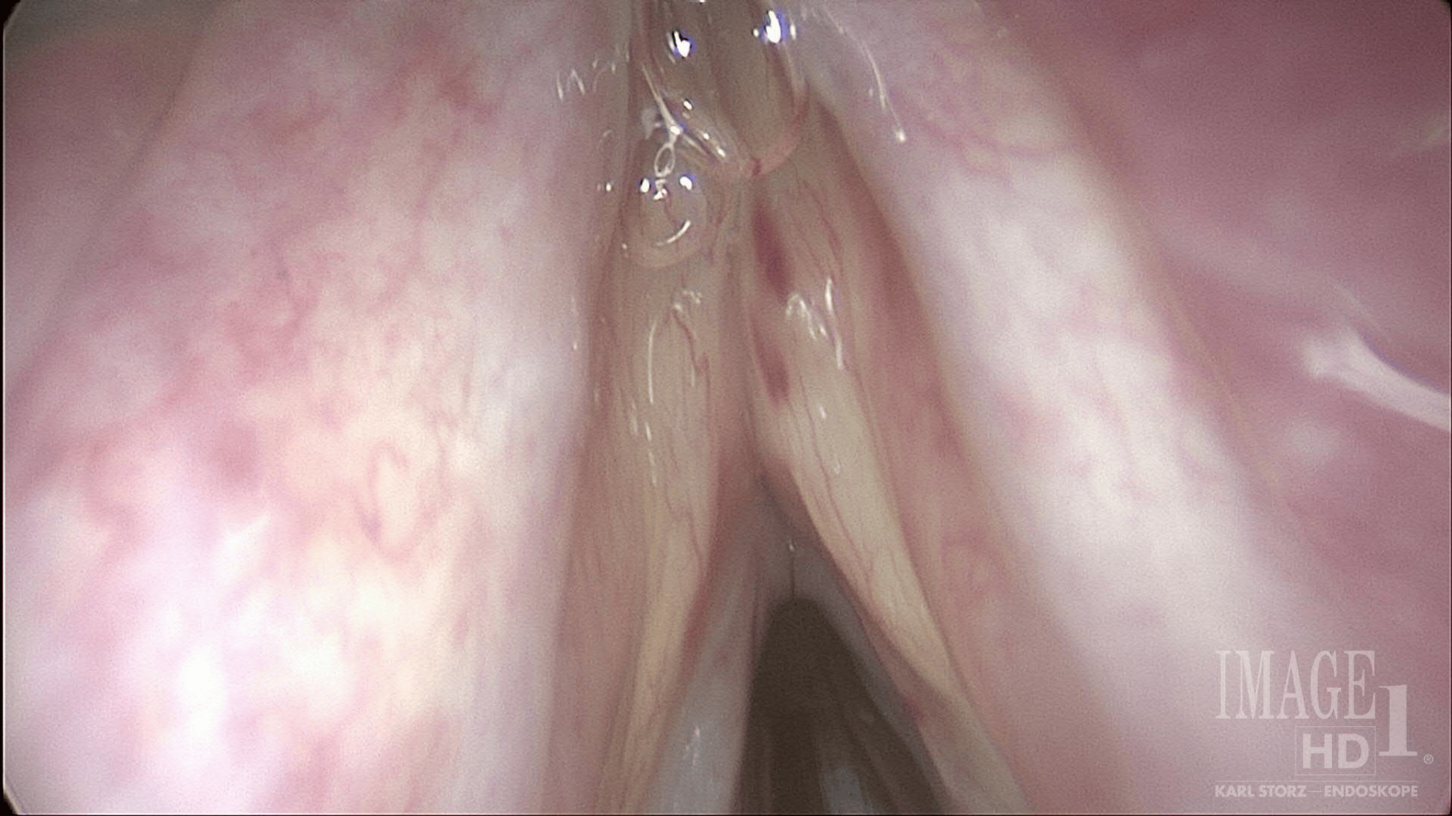
Bilateral vocal fold edema noted on laryngoscopy

A 60-year-old man presented to the Laryngology Clinic with two months of gradual voice fatigue and vocal strain. In addition, he reported solid food dysphagia. The Voice Handicap Index score was 22. His past medical history was notable for chronic, recurrent sinus infections and nasal obstructive symptoms exacerbated during air travel and allergy season. He was using Flonase and Astelin nasal sprays daily. The patient also had pulmonary and renal sarcoidosis, and his most recent imaging via CT demonstrated complete resolution of the sarcoid. Over the course of months, he was treated for sinonasal mucosal inflammation and sinusitis with budesonide nasal rinses twice, a steroid dose pack twice, azithromycin, and then mometasone rinses. While using the mometasone rinses, the budesonide and antihistamine nasal sprays were discontinued. The laryngeal examination was notable for bilateral true and false vocal fold edema and adynamic vocal fold vibration with muscle tension dysphonia (Figure 1).

Grade, roughness, breathiness, asthenia, strain (GRBAS) scale was G3 R3 B2 A1 S2. The average fundamental frequency was higher than the normal range. The measure of low-to-high spectral ratio was below the normal range, and the cepstral spectral index of dysphonia (CSID) was markedly increased. When combined with other measures of noise in the acoustic signal (jitter percentage, shimmer percentage, and noise-to-harmonic ratio), the laryngeal function studies support the diagnosis. The pitch range was higher than the normal limits, and the loudness range was decreased. Aerodynamic assessment revealed a decreased expiratory volume, markedly decreased maximum phonation time, markedly increased mean peak air pressure, and markedly increased phonation threshold pressure, indicating increased effort to produce phonation.

**Figure 2:**
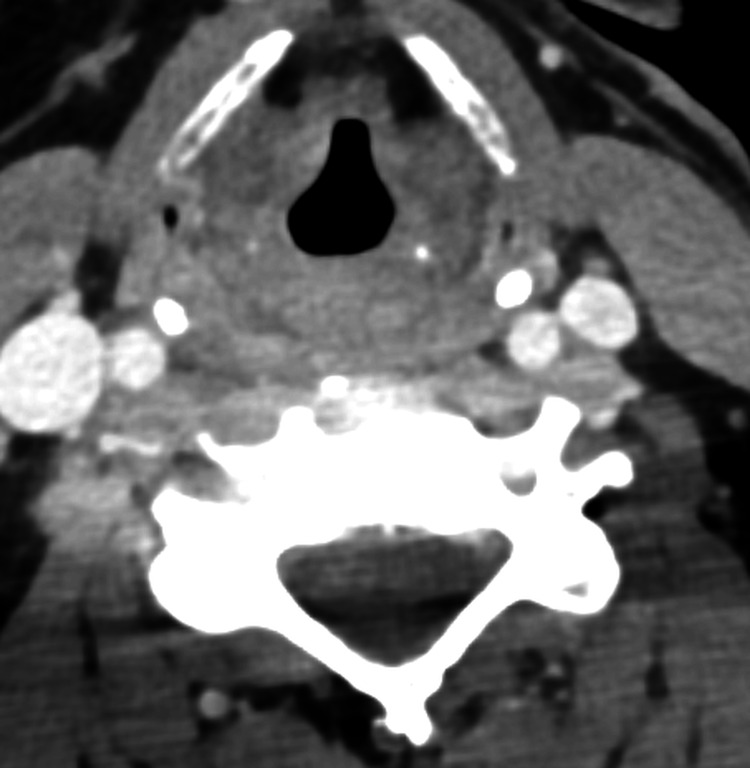
CT of the neck with contrast, which revealed edema involving the true and false vocal folds, aryepiglottic folds, and paraglottic fat

Investigations included CT of the neck with contrast, which revealed edema involving the true and false vocal folds, aryepiglottic folds, paraglottic fat, and associated mucosal hyperenhancement of the piriform sinuses (Figure 2).

He underwent a microdirect laryngoscopy with biopsy, which revealed benign hyperplastic squamous mucosa. Pathology was negative for dysplasia, malignancy, and noncaseating granulomas. The patient independently decided to discontinue nasal steroid rinses with immediate resolution of his laryngeal symptoms.

## Discussion

Nasal-steroid-rinse-induced laryngeal edema is uncommon. Despite how unusual this reaction may be, it has been noted in additional case reports and calls attention to the importance of vigilance when choosing these medications for treatment options. Current literature indicates that the off-label use of budesonide for local therapy in treating chronic sinusitis is widespread in practice, with studies demonstrating its safety for both short- and long-term use [6,7]. However, upon further investigation into the scientific literature on the efficacy, safety, and side effects of corticosteroids, the benefits and challenges associated with these treatments emphasize the need for a nuanced approach to their use and the importance of further research. Although specific research on the adverse effects of intranasal corticosteroids (INCS) on the vocal cords is limited, extensive literature exists on the inhaled form of corticosteroids, which cannot be overlooked given its direct impact on the larynx. Inhaled corticosteroids (ICS) are a cornerstone in managing chronic respiratory conditions such as asthma, but their use is not without side effects. A significant concern is dysphonia linked to the prolonged use of standard particle ICS [8]. Research indicates that smaller particle ICS, such as ciclesonide and beclomethasone, are less likely to cause vocal cord atrophy compared to standard-size particles found in medications such as budesonide-formoterol and fluticasone [8]. However, this study's conclusions were limited by a small sample size and the inclusion of patients with extensive histories of ICS usage, which, when removed from the analysis, rendered the findings statistically insignificant.

Despite advancements in ICS and INCS formulations, understanding their local side effects, particularly on the voice, remains limited. Newer ICS, such as ciclesonide and beclomethasone dipropionate, appear to be associated with a lower prevalence of dysphonia. Yet, the literature on this subject is sparse, and little progress has been made in understanding the mechanisms, prevention, and management of these side effects [9,10]. Adding to the complexity, a study found that ICS and INCS use significantly increases the risk of dysphonia, affecting up to 58% of patients [11]. This risk remains consistent across different routes of administration, including nasal, oral, and inhaled forms, as well as various corticosteroid types, indicating that the association between INCS use and dysphonia is a widespread phenomenon not limited to specific formulations. In addition to intranasal sprays, nasal corticosteroid rinses are commonly used to treat rhinosinusitis and nasal polyposis, offering comparable efficacy and safety profiles. This further suggests a shared risk of dysphonia as a potential adverse effect [12]. One plausible theory requiring further research is that prolonged corticosteroid use may lead to vocal cord atrophy, potentially resulting in secondary complications such as candida laryngitis [4]. This risk appears consistent across different inhaler types and corticosteroid medications, suggesting that the association between ICS use and dysphonia is a broad phenomenon not confined to specific drug formulations. In addition to inhaled forms, nasal corticosteroids are often used in treating rhinosinusitis and nasal polyposis and show similar efficacy and safety profiles as inhaled corticosteroids [12].

While corticosteroids, particularly in nasal forms, provide significant therapeutic benefits, their use also carries risks that require careful management. The observed association between corticosteroid use and dysphonia, along with variability in outcomes between INCS and nasal steroid rinses, highlights the need for further research to refine treatment strategies and minimize adverse effects [13]. This literature review highlights the importance of individualized treatment with corticosteroid therapies.

## Conclusions

While the benefits of nasal corticosteroid rinses in managing chronic rhinosinusitis and allergic rhinitis are well-documented, research on their atypical adverse effects remains limited. Cases of dysphonia and laryngeal edema, as highlighted here, underscore the need for further investigation into their safety profiles. These adverse effects, though uncommon, can significantly impact patients' quality of life and may require alternative management strategies. As treatment guidelines evolve, clinicians must stay informed about new advancements and potential risks to ensure optimal patient care. Expanding research into these less common outcomes is vital for deepening our understanding and shaping safer, more effective therapies for patients with chronic recurrent rhinosinusitis. This includes recognizing early signs of complications, such as bothersome voice changes associated with intranasal topical steroid use, to allow for timely intervention or discontinuation when necessary.
